# Nonuniform Effect of Carrier Separation Efficiency and Light Absorption in Type-II Perovskite Nanowire Solar Cells

**DOI:** 10.1186/s11671-017-1912-4

**Published:** 2017-03-01

**Authors:** Weiping Wang, Jialun He, Yiyan Cao, Lijing Kong, Xuanli Zheng, Yaping Wu, Xiaohong Chen, Shuping Li, Zhiming Wu, Junyong Kang

**Affiliations:** Department of Physics, Fujian Key Laboratory of Semiconductor Materials and Applications, Collaborative Innovation Center for Optoelectronic Semiconductors and Efficient Devices, Xiamen University, Xiamen, 361005 People’s Republic of China

**Keywords:** ZnO/CH_3_NH_3_PbI_3_ coaxial nanowires, Nonuniform effect, Carrier separation efficiency, Solar cell

## Abstract

Coaxial structures exhibit great potential for the application of high-efficiency solar cells due to the novel mechanism of radial charge separation. Here, we intensively investigate the nonuniform effect of carrier separation efficiency (CSE) and light absorption in perovskite-based type-II coaxial nanowire solar cells (ZnO/CH_3_NH_3_PbI_3_). Results show that the CSE rapidly decreases along the radial direction in the shell, and the value at the outer side becomes extremely low for the thick shell. Besides, the position of the main light absorption gradually moves to the outer side with the increase of the shell thickness. As a result, the external quantum efficiency shows a positional dependence with a maximal value close to the border of the nanowire. Eventually, in our case, it is found that the maximal power conversion efficiency of the solar cells reduces from 19.5 to 17.9% under the effect of the nonuniformity of CSE and light absorption. This work provides a basis for the design of high-efficiency solar cells, especially type-II nanowire solar cells.

## Background

Recently, the lead halide perovskite (CH_3_NH_3_PbX_3_,X = Cl, Br, I)-based solar cells (PSCs) have attracted considerable attention because of their high power conversion efficiencies (PCEs) and simple fabrication technique [1–5]. In previous studies, PSCs were generally fabricated by employing a similar structure to dye-sensitized solar cells with mesoporous-TiO_2_ as the electron transportation layer (ETL) [6–8]. Nowadays, many research interests turn to the planar architecture PSCs of ITO/hole transportation layer (HTL)/perovskite/ETL, which exclude the mesoporous-TiO_2_ layer. The reported PCEs are about 15% in this kind of cells [9–11]. With the purpose of further improving cell performance, many efforts, such as process modification and interface engineering, have been made [12, 13]. For example, Nie et al. fabricated planar solar cells with a PCE approaching 18% by using a hot-casting technique [12]; Zhou et al. boosted the cells with an average PCE up to 16% by using Yttrium-doped TiO_2_ as the ETL [13]. Compared with conventional film structure, coaxial structures have larger surface-area-to-volume ratio, longer light absorption length, and higher carrier separation efficiency (CSE) [14–17]. As such, coaxial structures may provide a great potential for the application of high-efficiency PSCs.

**Fig. 1:**
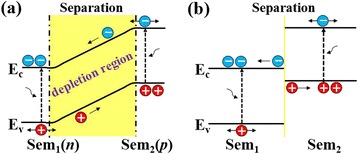
Carrier separation mechanisms. **a** p-n junction. (**b**) Type-II heterojunction

Generally, two kinds of coaxial structures are used for nanowire solar cells, i.e., p-n junction and type-II heterojunction [14–18]. As for the p-n junction cells, carriers are separated by the built-in electric field in the depletion region. As shown in Fig. 1a, if the nanowire is sufficiently thin, the depletion region will be comparable to the size of the nanowire, and the CSE can approach up to nearly 100%. However, for type-II heterojunction solar cells, the carrier separation is merely realized by the energy level difference at the interface, as shown in Fig. 1b. It means that only the carriers diffused to the interface can be separated, which results in the nonuniform CSEs inside the nanowires. Hence, in addition to the nanowire structure and the light absorption, the light distribution and the diffusion process of photo-generated carriers inside the nanowires will considerably impact cell performance [19–21]. Nevertheless, up to date, most of the studies put emphasis on the enhancement of light absorption by optimizing the design of the nanowire cells [22–24]; and there are very few investigations on the nonuniform effect of the CSE and light absorption in type-II nanowire cells.

In this work, we construct a kind of type-II coaxial perovskite nanowire (ZnO/CH_3_NH_3_PbI_3_) and intensively investigate the nonuniformity of CSE, light absorption, and external quantum efficiency (EQE) inside the nanowires by combining the semiconductor diffusion theory and finite-difference time-domain (FDTD) simulations [22–25]. Results demonstrate that the CSE rapidly decreases along the radial direction in the shell, which totally differs from that of the p-n junction nanowires. Besides, the light absorption inside the nanowires also shows a nonuniform feature. As a result, the EQE presents a positional dependence with a maximum value close to the border of the nanowire. Eventually, in our case, an ideal PCE of 19.5% is obtained in the nanowire with the shell thickness of ~60 nm, and this value decreases to 17.9% when considering the nonuniform effect of CSE and the light absorption. This work provides guidance on the design of high-efficiency solar cells.

## Methods

### Theory of CSE

**Fig. 2:**
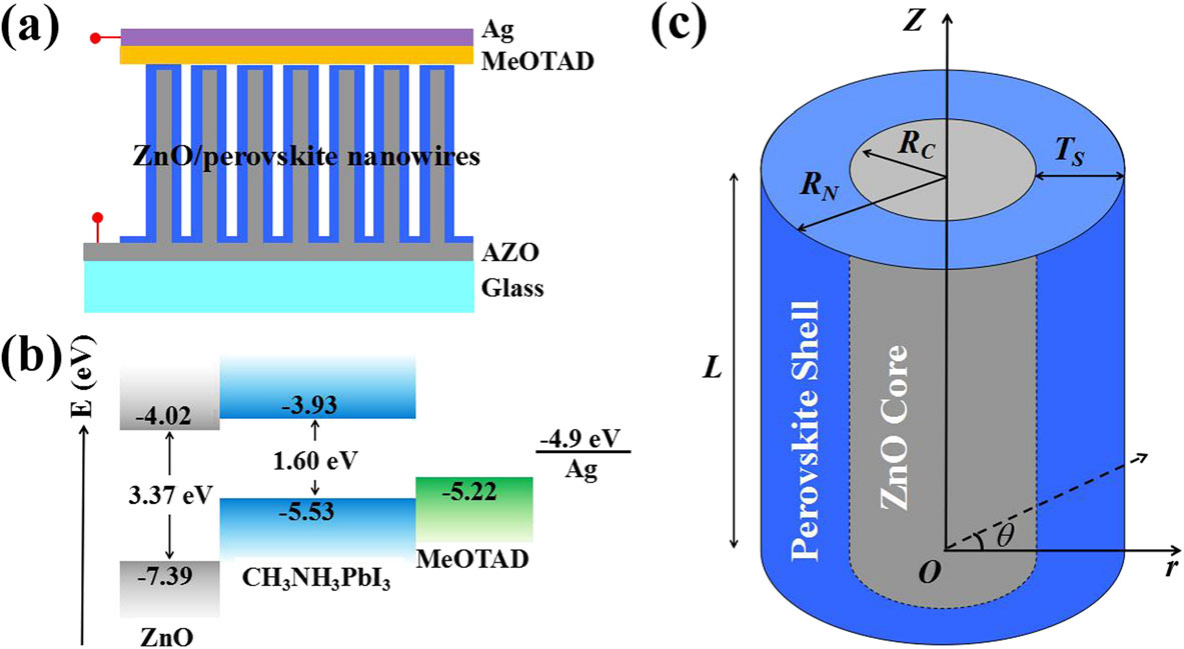
**a** Schematic diagram of a ZnO/CH_3_NH_3_PbI_3_ coaxial nanowires solar cell. **b** Energy-level alignment. **c** A model of single coaxial nanowire

Figure 2a shows a schematic diagram of ZnO/CH_3_NH_3_PbI_3_ nanowire solar cell, in which 2,2′,7,7′-tetrakis-(N,N-di-p-methoxyphenyl-amine)-9,9′-spirobifluorene (spiro-MeOTAD) and sliver are used as the HTL and the electrode, respectively. Figure 2b demonstrates the energy level diagram. A type-II energy alignment is formed at the interface between the ZnO and CH_3_NH_3_PbI_3_, which supports the separation of photo-generated carriers. To calculate the CSE of coaxial nanowire cells, a theoretical model is constructed. As shown in Fig. 2c, *R*_*C*_, *R*_*N*_, *T*_*S*_, and *L* represent the radius of the core, the radius of the whole nanowire, the shell thickness, and the length of the nanowire, respectively. When the light normally irradiates on the top surface of the nanowire, the holes (minority-carriers) in the core satisfy the continuity equation [25, 26],

1 $$ \frac{\partial \varDelta h(r)}{\partial t}=\frac{1}{q}\nabla \cdot {\overrightarrow{J}}_h-\frac{\varDelta h(r)}{\tau_h}+{g}_h, $$

where, *τ*_*h*_ is the lifetime of carriers, s*g*_*h*_ is the generation rate of the non equilibrium carriers, ${\overrightarrow{J}}_n$ is the current density, and *Δh*(*r*) is the distribution of excess hole concentration with respect to the equilibrium value. Assuming that all the carriers diffuse along the radial direction, namely, minority-carrier diffusion in the vertical direction is negligible [17], the steady-state Eq. (1) can be then derived as below,

2 $$ {D}_h\frac{\partial^2\varDelta h(r)}{\partial {r}^2}-\frac{\varDelta h}{\tau_h}+{g}_h=0, $$

where *D*_*h*_ stands for the hole-diffusion coefficient, which depends on the hole drift velocity and material temperature.

As the diffusion process reaches a balance, Eq. (2) should satisfy two boundary conditions:

(i) The increase of the photo-generated holes is equal to that of the decrease amount, which includes the recombination loss and the diffusion part (from core to shell). It can be expressed as,
  3 $$ 2\pi \cdot \frac{g_h{\tau}_h L{R_C}^2}{2}=2\pi \cdot {f}_{C\to S}\varDelta h(0){\tau}_h L{R}_C+2\pi \cdot \frac{L}{2}{\displaystyle {\int}_0^{R_C}\varDelta h(r) dr}, $$
  where *f*_*C* → *S*_ is the outflow rate of the holes at the interface. It is related to the band alignment of the two materials. Larger band offset leads to a greater *f*_*C* → *S*_.
(ii) The gradient of hole concentration is proportional to the outflow rate at the interface,
  4 $$ {\left.{D}_h\frac{\partial \varDelta h(r)}{\partial r}\right|}_{r={R}_C}={f}_{C\to S}\varDelta h(0). $$

Based on the above analysis, the hole concentration distribution in the core is obtained as follows,

5 $$ \kern1em \varDelta h(r)={\tau}_h{g}_h\left[1-\frac{f_{C\to S}{\tau}_h{e}^{-{R}_C/{L}_C}/2}{f_{C\to S}{\tau}_h \cosh \left({R}_C/{L}_C\right)+{L}_C \sinh \left({R}_C/{L}_C\right)}{e}^{R_C- r/{L}_C}-\frac{f_{C\to S}{\tau}_h{e}^{-{R}_C/{L}_C}/2}{f_{C\to S}{\tau}_h \cosh \left({R}_C/{L}_C\right)+{L}_C \sinh \left({R}_C/{L}_C\right)}{e}^{r-{R}_C/{L}_C}\right],\kern1em $$

where ${L}_C=\sqrt{D_h{\tau}_h}$ is the hole-diffusion length in the core. Considering the relatively large band offset at the interface, *f*_C → S_ is assumed to be infinite, then Eq. (4) is simplified as,

6 $$ \varDelta h(r)={\tau}_h{g}_h\left[1-\frac{ \cosh \left(\frac{r}{L_C}\right)}{ \cosh \left(\frac{R_C}{L_C}\right)}\right],\kern0.5em 0\le r<{R}_C, $$

To simplify the discussion, we define the ratio of the thickness of the material to the carrier diffusion length as *k*_*i*_, (*i* = *c*, *s*), where the subscript c and s represent the core and the shell, respectively. Consequently, the CSE of the core is derived as below,

7 $$ {\mathrm{CSE}}_C(r)=\frac{ \cosh \left(\frac{r}{L_C}\right)}{ \cosh \left(\frac{R_C}{L_C}\right)}=\frac{ \cosh \left(\frac{r}{L_C}\right)}{ \cosh \left({k}_C\right)},\kern0.5em 0\le r<{R}_C. $$

Similarly, the electron concentration distribution and CSE of the shell are expressed as,

8 $$ \varDelta e(r)={\tau}_e{g}_e\left[1-\frac{ \cosh \left(\frac{r-{R}_N}{L_S}\right)}{ \cosh \left(\frac{T_S}{L_{\mathrm{S}}}\right)}\right]={\tau}_e{g}_e\left[1-\frac{ \cosh \left(\frac{r-{R}_C}{L_S}-{k}_S\right)}{ \cosh \left({k}_S\right)}\right], $$

9 $$ {\mathrm{CSE}}_S(r)=\frac{ \cosh \left(\frac{r-{R}_C}{L_S}-{k}_S\right)}{ \cosh \left({k}_S\right)},\kern0.5em {R}_C< r\le {R}_N. $$

### Optical Simulation

The light absorption and its distribution in coaxial nanowires were stimulated by using the software *FDTD Solutions* from Lumerical Solutions. In the simulation, an aluminum-doped zinc oxide (AZO) transparent conducting layer with the thickness of 500 nm was deposited on the 1000-nm-thick glass and used as the substrate. Coaxial nanowire array was constructed with a period of 400 nm above the substrate. The length *L* and the core radius *R*_*C*_ of nanowires were fixed to be 1000 nm and 25 nm, respectively. The shell thickness varied from 25 to 150 nm. The optical parameters of ZnO, AZO, and perovskite material (CH_3_NH_3_PbI_3_) were acquired from Ref. [22] and Ref. [27], respectively, and the background index is set to 1. The diffusion length of perovskite material is set to 130 nm based on the experimental result in Ref. [28]. During the simulation, light normally irradiated on the top of nanowires, and all the results were normalized with the standard one sun AM 1.5G illumination (100 mW/cm^2^).

### Calculation of EQE and PCEs

Ideally, assuming that each absorbed photon generates an electron-hole pair, and all the electron-hole pairs can be separated and extracted out of the nanowires, the photo-generated short-circuit current *I*_*SC*_ can be calculated by weighting the incident solar spectrum (spectral power *P*_*AM 1.5G*_(*λ*)) with the absorption as below [29],

10 $$ {I}_{S C}={A}_{S C}\frac{e}{hc}{\displaystyle {\int}_{R_C}^{R_S}{\mathrm{CSE}}_S(r)\left[{\displaystyle {\int}_{\lambda =0}^{\lambda_g=\frac{hc}{E_g}}\lambda \alpha \left( r,\lambda \right){P}_{A M1.5 G}\left(\lambda \right) d\lambda}\right]} d r, $$

where *α*(*r*, *λ*) is the *r*-dependent absorptivity and *A*_*SC*_ is the macroscopic area of a nanowire array solar cell constituting a large integer number of unit cells.

Considering the nonuniformity of CSE and light distribution inside the nanowires, the EQE at different radial position *r* is different, which can be calculated by the following formula,

11 $$ \begin{array}{l} EQE(r)=\frac{2\pi rdr\Big[ CS{E}_S(r){\displaystyle {\int}_{\lambda =0}^{\lambda_g=\frac{hc}{E_g}}\frac{\lambda}{hc}\alpha \left( r,\lambda \right){P}_{AM1.5 G}\left(\lambda \right) d\lambda \Big]}}{2\pi rdr{\displaystyle {\int}_{\lambda =0}^{\lambda =\infty}\frac{\lambda}{hc}{P}_{AM1.5 G}\left(\lambda \right) d\lambda}}\\ {}=\frac{CS{E}_S(r){\displaystyle {\int}_{\lambda =0}^{\lambda_g=\frac{hc}{E_g}}\lambda \alpha \left( r,\lambda \right){P}_{AM1.5 G}\left(\lambda \right) d\lambda}}{{\displaystyle {\int}_{\lambda =0}^{\lambda =\infty}\lambda {P}_{AM1.5 G}\left(\lambda \right) d\lambda}}.\end{array} $$

Theoretically, the ultimate efficiency (*UE*) of a solar cell can be expressed as

12 $$ U E=\frac{E_g\cdot {I}_{SC}/ e}{100\  mW/ c{m}^2\cdot {A}_{SC}}, $$

where *E*_*g*_ represents the band offset between the ZnO and the perovskite material. According to the energy level in Fig. 2b, the value of *E*_*g*_ is 1.51 eV. Based on Skockley and Queisser’s theory [30], the PCE of a solar cell can be derived as follows,

13 $$ P C E = \frac{V_{OC}\cdot {I}_{SC}/{A}_{SC}\cdot \mathrm{FF}}{100\  mW/ c{m}^2}= U E\cdot v\cdot F F. $$

Here, *v* is the ratio of open circuit voltage (*V*_*OC*_) to *E*_*g*_ */e*, and FF is the fill factor. According to equations (3.19) and (5.5) in Ref. [30], the maximal *V*_*OC*_ and FF at room temperature (300 K) can be obtained, and their values are 1.24 V and 90%, respectively.

## Results and Discussion

**Fig. 3:**
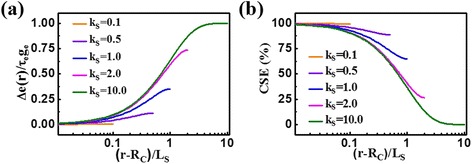
Distributions and CSEs of non equilibrium carriers in the shell layer. **a** The concentration distributions. **b** CSEs

As is known, light absorption and CSEs are the key factors affecting PCE. Considering that most of the light is absorbed in the shell due to the narrow bandgap of shell materials [22–24], we put emphasis on the investigation of the relevant characteristics in this layer. Figure 3a shows the concentration distributions of the nonequilibrium carriers with different *k*_*s*_ under uniform light irradiation. It can be seen that the concentration is very low (approximately zero) in the entire shell layer when *k*_*s*_ is small (i.e., a thin shell); with the increase of *k*_*s*_, the concentration at the interface almost remains unchanged; however, it gradually increases in the outer shell. Figure 3b reveals the corresponding CSE results, showing significant dependence on the shell thickness (or *k*_*s*_) that thinner shell generates higher CSE. The maximal CSE approaches almost 100% for the *k*_*s*_ of 0.1, and it is still over 60% at the outer surface when *k*_*s*_ increases to 1.0. Note that the CSE at the outer side becomes extremely low as *k*_*s*_ exceeds a certain value and is close to zero for the *k*_*s*_ of 10.0. This is because that, in this case, the carriers at the outer side are difficult to diffuse to the interface for separation. Thus, a thin shell is beneficial for the carrier separation. On the other hand, a thin shell might be unfavorable for the light absorption. Therefore, shell thickness is an important parameter in designing and fabricating high-efficiency solar cell.

**Fig. 4:**
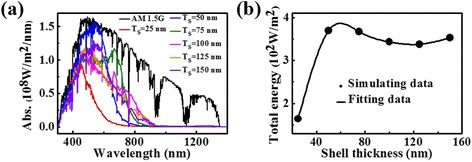
Absorption characterization. **a** Absorption spectra of coaxial nanowires with different radii under AM 1.5G illumination (100 mW/cm^2^). **b** total absorbed energies of different coaxial nanowires

To investigate the influence of the shell thickness on the light absorption, we simulated the absorption spectra of coaxial nanowires with different shell thickness under AM 1.5G illumination (100 mW/cm^2^). As shown in Fig. 4a, the main absorption of all the nanowires occurs in visible region with a threshold wavelength of about 800 nm, corresponding to the bandgap (1.60 eV) of CH_3_NH_3_PbI_3_. The absorption peaks and intensities vary with the shell thickness *T*_*S*_. The absorption peaks first redshift and then blueshift with the increase of *T*_*S*_, but the total absorbed energies have a dramatic increase at the beginning and then tend to be saturated. The strongest absorption appears with the shell thickness of ~60 nm. This behavior may be related to the quantity of the absorbing material and the number of optical guided modes in the nanowires [24, 31–34]. When the nanowire is thin, the incident light, especially in the long-wavelength regime, easily penetrates without significant interaction, resulting in a poor absorption. With the increase of *T*_*S*_, the increased shell material and numbers of guided modes are both helpful to improve the light absorption, which causes the redshift of absorption peak. However, further increasing the *T*_*S*_ will lead to energy loss due to the increased light reflection and transmission [34] and results in the blueshift of absorption peak. It is believed that the light absorption can be further enhanced by optimizing the structure and the dimension of coaxial nanowires.

**Fig. 5:**
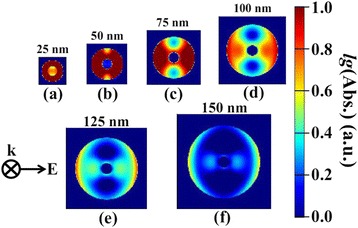
The distribution of the total absorption energy in x-y plane for nanowires with different shell thickness. Integrated wavelength covers from 300 nm to 1000 nm. *T*_*S*_: **a** 25 nm, **b** 50 nm, **c** 75 nm, **d** 100 nm, **e** 125 nm, and **f** 150 nm

It is clear that CSE varies along the radial direction in type-II coaxial nanowires according to the analysis in Fig. 3. Hence, the light distribution in nanowires will significantly affect the PCEs of cells. To reveal the intrinsic influence, we further calculated the distribution of total absorption energy in x-y plane. As shown in Fig. 5, for all the cases, the light unevenly propagates inside the nanowire, and most of the light energy is absorbed by the perovskite shell due to its narrow bandgap. Note that the absorbed energy does not uniformly distribute in the shell layer; moreover, the maximum absorption region gradually moves to the outer side with the increase of shell thickness. This is attributed to the higher refractive index of the shell than that of the air, which results in a strong reflection at the shell/air interface. Nevertheless, as suggested above, the farther the photo-generated carriers are away from the interface, the lower the CSE is. Therefore increasing shell thickness that does not necessarily enhance PEC.

**Fig. 6:**
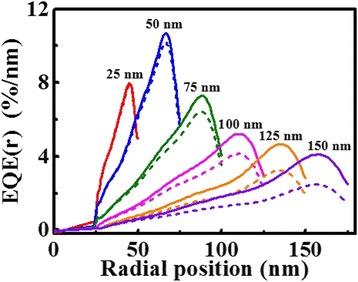
EQEs per unit radial length at different positions for the nanowires with different shell thickness under AM 1.5G illumination. *Solid lines* and *dash lines* represent the results of the nanowire with *L*_*S*_ = ∞ and *L*_*S*_ = 130 nm [28], respectively. Integrated wavelength covers from 300 to 1000 nm

Taking the nonuniformity of CSE and light distribution inside the nanowires into account (seeing Fig. 3), we obtained the EQE per unit radial length at different positions by Eq. (7) (diffusion length *L*_*S*_ is set to 130 nm). For comparison, we also calculated the corresponding EQEs of the nanowires without considering the effect of nonuniform CSE (diffusion length *L*_*S*_ is infinite). As shown in Fig. 6, the EQE in shell layers are much larger than that in ZnO core layers owing to the stronger light absorption. In addition, for all the nanowires, the peak positions of EQEs are close to that of the outer sides, which is similar to the phenomenon of the light absorption distributions shown in Fig. 5. The peak values of EQEs are quite different for different nanowires. The nanowire with a shell thickness of 50 nm shows a maximal EQE at the position with the radius of 66 nm. It should be pointed out that, with the consideration of the nonuniform CSE in type-II coaxial nanowires, all the EQEs reduce in varying degrees, and the decrease become more distinguished for the thicker nanowire. This means that there is an optimized shell thickness for designing high-efficiency solar cells.

**Fig. 7:**
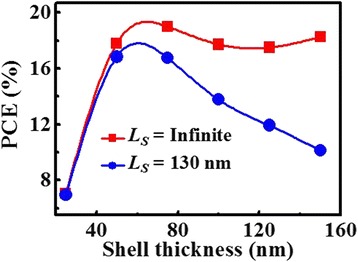
PCEs of ZnO/CH_3_NH_3_PbI_3_ solar cells

Figure 7 shows the dependence of PCEs on the shell thickness. For the nanowire with an infinite diffusion length, its PCE initially increases with the increasing shell thickness and reaches the peak value of 19.5% for 60 nm shell thickness. The PCE decreases as the shell thickness increases beyond 60 nm. While taking the actual nonuniform CSE into account (*L*_*S*_ = 130 nm) [28], the PCE decreases in varying degrees with the shell thickness, and moreover, the decreasing becomes more evident for the thicker shell. In this case, the peak value reduces from 19.5 to 17.9%. Notably, this work focuses on the effect of CSE and light absorption on PCE, and the PCE of 17.9% could be improved by optimizing other parameters, such as nanowire length and core radius. In short, when designing or fabricating type-II coaxial nanowire solar cells, it is necessary to comprehensively evaluate the influence of nonuniform CSE, light absorption distribution, and geometrical dimensions on cell performance.

## Conclusions

In conclusion, we deeply investigate the nonuniformity of the CSE, the light absorption, and the EQEs at different positions inside type-II ZnO/CH_3_NH_3_PbI_3_ nanowires, and their influence on PCEs of nanowire solar cells by combining the semiconductor diffusion theory and FDTD simulations. Results show that the CSE rapidly decreases along the radial direction in the shell, and the value at the outer side becomes extremely low for the thick shell. The light absorption intensity varies with shell thickness. Meanwhile, the absorbed energy does not uniformly distribute in the shell layer, and the peak position gradually moves towards the outer side with the increase of the shell thickness. As a result, the peak positions of EQEs are close to the outer side, and the maximal EQE is obtained in the nanowire with the shell thickness of 60 nm. Finally, we calculate the PCEs of coaxial nanowire solar cells. It is found that the nonuniform CSE and light absorption in type-II nanowires will result in a decrease of PCE, and the decreasing becomes more evident for the nanowire with the thicker shell. In the case with the diffusion length of 130 nm, the maximal value reduces from 19.5 to 17.9%. Although this work focuses on the ZnO/CH_3_NH_3_PbI_3_ coaxial nanowire cell, this method can be applied to other wide-bandgap semiconductor/perovskite type-II nanowire cells. In all, this work provides guidance on the design of high-efficiency solar cells, especially the type-II coaxial nanowire solar cells.
